# Cross-sectional associations between mental health indicators and social vulnerability, with physical activity, sedentary behaviour and sleep in urban African young women

**DOI:** 10.1186/s12966-022-01325-w

**Published:** 2022-07-10

**Authors:** Catherine E. Draper, Caylee J. Cook, Stephanie Redinger, Tamsen Rochat, Alessandra Prioreschi, Dale E. Rae, Lisa J. Ware, Stephen J. Lye, Shane A. Norris

**Affiliations:** 1 SAMRC-Wits Developmental Pathways for Health Research Unit, Faculty of Health Sciences, University of the Witwatersrand, Johannesburg, Gauteng South Africa; 2 DSI-NRF Centre of Excellence in Human Development, University of the Witwatersrand, Johannesburg, South Africa; 3 Health through Physical Activity, Lifestyle and Sport Research Centre & Division of Physiological Sciences, Department of Human Biology, Faculty of Health Sciences, University of Cape Town, Cape Town, South Africa; 4 Lunenfeld-Tanenbaum Research Institute, Sinai Health System, Toronto, Canada; 5 Departments of Obstetrics and Gynecology, Physiology and Medicine, University of Toronto, Toronto, Canada; 6 Global Health Research Unit, School of Human Development and Health, University of Southampton, Southampton, UK

**Keywords:** Low- and middle-income country, Physical activity, Sedentary behaviour, Sleep, Mental health

## Abstract

**Background:**

Relationships between mental health and multiple health behaviours have not been explored in young South African women experiencing social constraints. The aim of this study was to identify associations between mental health indicators and risk factors with physical activity, sedentary behaviour, and sleep, amongst young women living in Soweto, a predominantly low-income, urban South African setting.

**Methods:**

For this cross-sectional study, baseline measurements for participants (*n* = 1719, 18.0–25.9 years old) recruited for the Healthy Life Trajectories Initiative were used including: physical activity, sedentary behaviour (sitting, screen and television time), sleep (duration and quality), depression and anxiety indicators, emotional health, adverse childhood experiences, alcohol-use risk; social vulnerability, self-efficacy, and social support.

**Results:**

Multiple regression analyses showed that depression (β = 0.161, *p* < 0.001), anxiety (β = 0.126, *p* = 0.001), adverse childhood experiences (β = 0.076, *p* = 0.014), and alcohol-use risk (β = 0.089, *p* = 0.002) were associated with poor quality sleep. Alcohol-use risk was associated with more screen time (β = 0.105, *p* < 0.001) and television time (β = 0.075, *p* < 0.016). Social vulnerability was associated with lower sitting time (β = − 0.187, *p* < 0001) and screen time (β = − 0.014, *p* < 0.001). Higher self-efficacy was associated with more moderate- to vigorous-intensity physical activity (β = 0.07, *p* = 0.036), better-quality sleep (β = − 0.069, *p* = 0.020) and less television time (β = − 0.079, *p* = 0.012). Having no family support was associated with more sitting time (β = 0.075, *p* = 0.022). Binomial logistic regression analyses supported these findings regarding sleep quality, with anxiety and depression risk doubling the risk of poor-quality sleep (OR = 2.425, *p* < 0.001, OR = 2.036, *p* = 0.003 respectively).

**Conclusions:**

These findings contribute to our understanding of how mental health indicators and risk factors can be barriers to health behaviours of young women in Soweto, and that self-efficacy and social support can be protective for certain of these behaviours for these women. Our results highlight the uniqueness of this setting regarding associations between mental health and behaviours associated with non-communicable diseases risk.

**Supplementary Information:**

The online version contains supplementary material available at 10.1186/s12966-022-01325-w.

## Introduction

Prior to the COVID-19 pandemic in 2020, mental disorders were well established to be a leading cause of the global health-related burden, with depression and anxiety being leading contributors to this burden, along with harmful alcohol use. Substantial evidence suggests this burden will worsen post-pandemic [1]. In 2018, the United Nations expanded their non-communicable diseases (NCDs) focus to a five-by-five approach, formally including mental disorders and mental health conditions amongst the big five NCDs (in addition to cardiovascular diseases, diabetes, cancer, and chronic respiratory diseases), while also identifying five important risk factors (tobacco use, unhealthy diet, physical inactivity, harmful alcohol use, and high levels of environmental risks, including childhood adversity) associated with most NCDs, including mental health [2]. This five-by-five approach provides some rationale for investigating the relationship between mental disorders and mental health conditions and risk factors for these disorders and conditions, and how these relationships are evident in different populations. In low- and middle-income countries (LMICs), this burden of common mental health problems and harmful alcohol use exists alongside other high burdens of infectious diseases and NCDs [3]. Despite this, prevention, interventions and treatment are limited in LMICs [4], and environmental challenges in these countries contribute substantially to disease burden, particularly for younger adults, where opportunities for prevention could have substantial impact across the lifecourse [5].

Amongst adults in LMICs, there is a growing body of research on the relationship between mental health and physical activity [6–10], sedentary behaviour [8, 11, 12], and sleep [10, 13]. These studies have reported on cross-sectional associations, and therefore do not clarify causality to answer questions about whether poor mental health leads to poor health behaviours, or whether healthy behaviours lead to good mental health. There is however a growing body of research to better understand how mental health (as a big five NCD) is related to certain risk factors listed in the five-by-five approach. In 2020, the World Health Organization released revised evidence-based guidelines on physical activity and sedentary behaviour in adults that recognise the role of physical activity in promoting mental health and reducing NCDs [14]. Furthermore, there is evidence for the protective nature of physical activity against mental health conditions, and as a treatment for symptoms of mental health conditions in adults [15–17] and youth [18–22]. Evidence also exists for the inverse relationship between sedentary behaviour and mental health in adults [23, 24] and adolescents [22, 25, 26]. The bidirectional relationship between sleep and mental health (i.e. adequate, good quality sleep contributes to good mental health, and good mental health contributes to better, adequate sleep) is well-established [27–29], with symptoms of depression and anxiety being clearly associated with sleep duration, quality and timing [30–32]. Younger women appear to be particularly vulnerable [33, 34], and there is a positive relationship between meeting sleep duration recommendations and mental health for children and adolescents [22]. More recent research has documented associations between sleep duration and sleep quality, and mental health conditions among pregnant women [35], and harmful alcohol use (a risk for poor mental health) in women more generally [36]. A recent study on sleep in young adults in the United Kingdom found sleep quality to be the strongest independent predictor of mental health in women [37].

### Healthy life trajectories initiative

The Healthy Life Trajectories Initiative (HeLTI) is a multi-country study aimed at establishing a programme of research to generate evidence on intervening to optimise young women’s physical and mental health, and to establish healthier trajectories for themselves and potential future offspring, including reduced risk for NCDs [38]. As part of HeLTI, it is essential to understand NCD risks in young women, and how these might be mitigated through health behaviour change as well as mental health support.

The evidence presented above contributes to the rationale for examining the relationship between mental health indicators and health behaviours, namely physical activity, sedentary behaviour, and sleep, given that research has not been conducted to investigate these relationships with young women in South Africa. Furthermore, research has not been conducted to examine these relationships alongside other common risk factors for poor mental health, such as harmful alcohol use, adverse childhood experiences, and social vulnerability in young South African women. The relationships between sleep and mental health indicators are also poorly understood in this age group and population, although preliminary data are emerging describing the relationship between sleep duration and metabolic risk in South African women [39, 40]. In contrast to risk factors for poor mental health, self-efficacy and social support can be considered protective factors for healthy behaviours. Self-efficacy is considered a key determinant of behaviour change, and social support is a recognised behaviour change technique [41]. While the importance of self-efficacy and social support have been highlighted in the context of sexual behaviours and HIV in young South Africans [42–45], they have not been explored in relation to physical activity, sedentary behaviour, and sleep in the context of young women’s mental health in South Africa.

Soweto, the South African study site for HeLTI, is a predominantly low-income, urban setting in Johannesburg. In Soweto, young women are vulnerable to numerous mental health risks in their social and economic environment, including social injustice, gender-based violence and other traumatic events, and limited educational and employment opportunities [46]. In this setting, exposure to adverse childhood experiences is high [47], and the prevalence of antenatal anxiety and depression was been found to be 15 and 27% respectively [48]. Formative qualitative work conducted for HeLTI has highlighted the socioeconomic constraints experienced by these young women, and their need for mental health support [49]. These social constraints make it difficult for young women to prioritise their own health when making choices regarding their physical activity and dietary behaviour [50] in these circumstances [51, 52]. This is especially relevant since women in Soweto face numerous risks for other NCDs, including overweight and obesity [53], unhealthy diet [54–56], high sedentary behaviour [57, 58], and physical inactivity in late adolescence [59].

In previously reported findings from the HeLTI trial pilot data (~ 1600 women aged 18–25 years), 33% of women were classified as food insecure, 20% were at risk of being food insecure, and 44% were overweight/obese [60]. Most women met physical activity guidelines (based on self-report), but the majority of this activity was accumulated through transport- or work-related activity; less than half of the women reported any leisure time physical activity [61]. In this same sample, using an adapted social vulnerability index 27% of the total sample were classified as socially vulnerable, increasing to 44% for women with one child, and 64% for women with more than one child [46].

### Aim and hypotheses

To address the research gaps mentioned above, and in light of the multiple risks to the physical and mental health of young women in Soweto, the aim of this cross-sectional study was therefore to investigate the relationships between mental health indicators (depression, anxiety, emotional health), risk factors (adverse childhood experiences, harmful alcohol use, social vulnerability) and protective factors relating to health behaviour change (self-efficacy and social support), with health behaviour outcomes (physical activity, sitting, screen and TV time, and sleep duration and quality) amongst young women living in Soweto. This study was intended to generate initial evidence on cross-sectional associations, which could then provide direction for future research to further investigate the relationships between mental health indicators, risk, and protective factors in greater detail.

**Fig. 1:**
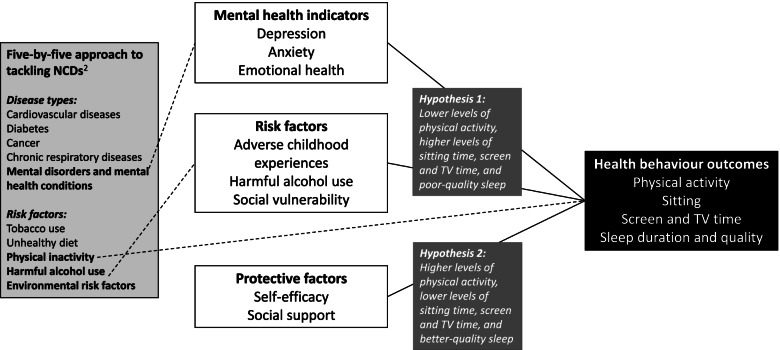
Study conceptual model

Given the Soweto context described above, along with the evidence presented earlier on the relationship between poor mental health and poor health behaviours in other LMICs, we hypothesised that similar associations would be observed amongst young women in Soweto. Specifically, we hypothesised that for young women in this setting, poor mental health indicators and increased mental health risk, indicated in the conceptual model in Fig. 1 below (identifying links with the five-by-five approach mentioned above), would be associated with lower levels of physical activity, higher levels of sitting time, screen and TV time, not meeting sleep duration guideline, and poor-quality sleep. In addition, it was hypothesised that higher levels of self-efficacy and social support would be associated with higher levels of physical activity, lower levels of sitting time, screen and TV time, meeting sleep duration guideline, and better-quality sleep. By addressing these hypotheses, we hope to contribute to answering the following research questions: if young women in Soweto have poor mental indicators, are they less likely to engage in healthy behaviours; and are they more likely to engage in these healthy behaviours if they have higher self-efficacy and more social support?

## Methods

### Participants

Participants were recruited as part of a survey for HeLTI, which was conducted in approximately 20,000 households in Soweto to characterise the density, composition, and socioeconomic status of these households [62]. The large sample size was intended to address the risk of selection bias. Young women (18.0–25.9 years of age) from these households were invited to take part in the next phase of HeLTI, which involved baseline testing for the HeLTI trial. Exclusion criteria (apart from age) included: diagnosis of type-I diabetes, cancer or epilepsy; intellectual disability that impeded informed consent; pregnancy at the time of the baseline assessment; and not able or willing to provide consent. The cross-sectional data presented in this paper comprise baseline measurement data from 1719 young women who participated in the pilot phase of the HeLTI trial. The study was approved by the Human Research Ethics Committee (Medical) at the University of the Witwatersrand (ref.: M171137, M1811111). All participants gave written informed consent for their participation in the study.

### Data collection and measures

Baseline data were collected from individual women at the research centre (at Chris Hani Baragwanath Academic Hospital) in Soweto (June 2018 – July 2019). Data collection was done using the REDCap electronic data capture tool [63] and involved a set of interview-administered questionnaires and physical measurements. Building on previous research evidence, and to align with the study conceptual model and address our hypotheses, relevant health behaviour outcomes were selected from this data set as dependent variables, and mental health indicators, risk and protective factors were selected as independent variables. Other outcomes from this pilot baseline dataset have been reported elsewhere [46, 60, 61].

#### Health behaviours

The Global Physical Activity Questionnaire (GPAQ) was used to assess self-reported moderate- to vigorous-intensity physical activity (MVPA, hours per week), sitting time (hours per week), screen time and TV time (hours per day) [64]. The Pittsburgh Sleep Quality Index (PSQI) questionnaire was used to assess sleep quality and duration, generating a continuous global PSQI score (higher scores indicating worse quality sleep) and a binary variable for poor sleep quality (PSQI global scores > 5) [65]. Participants were classified as meeting the physical activity guideline if they reported at least 150 minutes of MVPA per week and/or 75 min of vigorous activity per week [14], and meeting the sleep guideline if they reported between 7 and 9 hours of sleep per night [66]. Descriptive data from the GPAQ, as well as individual (excluding mental health) and community level predictors of these behaviours have been reported elsewhere [61]. While self-reporting of behaviours increases the risk of recall and social desirability biases, the use of measurement tools that have previously shown to be valid and reliable was an attempt to overcome these potential biases. Body mass index (BMI) was calculated from the direct measurement of height (to the nearest 0.1 cm, using a Holtain wall-mounted stadiometer) and weight (to the nearest 100 g using SECA scales) repeated three times, using standard protocols.

#### Mental health indicators

The 9-item Patient Health Questionnaire (PHQ-9) was used to assess probable depression (cut off of ≥10 for depressive symptoms, binary outcome variable) [67], and the Generalised Anxiety Disorder Scale (GAD-7) for probable anxiety (cut off of ≥10 indicating moderate to severe symptoms of anxiety, binary outcome variable) [67]. A happiness self-rating scale was used to obtain an indicator of emotional health (1–5 scale, 1 = very unhappy, 5 = very happy). Participants were also asked to indicate if they were taking medication for depression.

#### Mental health risk factors

Adverse childhood experiences were assessed using the Adverse Childhood Experiences (ACEs) Questionnaire (continuous score, higher score indicator higher number of adverse experiences) [68]. The World Health Organization Alcohol Use Disorders (WHO-AUDIT) Test was used to assess alcohol use risk (cut off of ≥8 for at-risk alcohol-use, binary outcome variable) [69]. Social vulnerability was assessed using a Social Vulnerability Index (SVI) adapted for use in South Africa, using this baseline dataset [46]. This SVI was based on socioeconomic status (household assets, employment, education); household composition and disability (household age, disability); and housing and transportation (housing type, household density, car ownership). One point was allocated for each of the indicators (range 0–8), and a participant was classified as socially vulnerable if their total score was in the 90th centile for the group (≥4) [46].

#### Mental health protective factors

Self-efficacy was assessed using the General Self-Efficacy Scale (GSE, continuous score, higher score indicator higher self-efficacy) [70]; and social support was assessed using a locally adapted set of questions [71] (family and partner, continuous score, higher score indicator higher level of support).

### Data analyses

Data were analysed using SPSS version 27.0 for Mac (SPSS Inc., Chicago, IL, USA). Normality of the data was checked using the Shapiro-Wilk test, and data were summarised and presented as mean ± standard deviation (SD), median (interquartile range, IQR), or number and frequency (%). Bivariate correlations were used to test for associations between variables. While leisure time physical activity may have been more informative in these analyses, previous findings indicated that less than half of this sample participated in any leisure time MVPA [61]. Since no significant associations were identified with leisure time MVPA when this was included in the regression analyses, total MVPA was used as the indicator of physical activity.

Data exploration indicated that the assumptions for multiple regression and binomial logistic regression were met for the analytic sample. ﻿While there were occasional minor deviations from normality (per Shapiro–Wilk tests), these were not extreme (as indicated by z_skewness_ statistics < 3). Multiple regression models were used to examine the associations between mental health indicators, risk and protective factors, and health behaviours (hours per week of total MVPA and total sitting time, hours per day of screen time and TV time, and global PSQI score). Binomial logistic regression models were used to examine the associations between exposures (mental health indicators, risk and protective factors) and outcomes (not meeting guidelines for MVPA and sleep, and being classified as having ‘poor quality sleep’ by the PSQI, to align with the stated hypotheses). Age and BMI were included in the regression models, but these are not discussed in this paper, since they have previously been reported for this sample [61]. Due to the sensitivity of some of the data collected, participants were not obliged to answer all the questions if they felt uncomfortable. Data imputation was not deemed appropriate; participants with complete data were therefore included in the regressions. Included and excluded participants for all main outcome variables did not differ significantly in terms of the SVI classification, which indicates that missing data was unlikely due to participants’ sociodemographic characteristics (encompassed by the variables included in the SVI).

## Results

Participant characteristics for the variables of interest are presented in Table 1 (including the valid sample size available for each variable). Of the participants included in these analyses (mean age = 21.2 ± 2.2y, median = 21 [19–23] y, *n* = 1593). More participants met the physical activity guideline (86.3%), compared to those meeting the sleep guideline (47.9%). A relatively small percentage (7.9%) were classified as being short sleepers (< 7 hours per night), while 44.1% were classified as being as long sleepers (≥9 hours per night); a third (33.4%) of participants were classified as having poor quality sleep. Most participants reported having a cell phone (97.9%) and TV (91.6%); 40.8% reported having a computer or laptop. A quarter of participants were classified as socially vulnerable and a similar number (24.2%) were at risk for harmful alcohol-use, while fewer were classified as having depression (18.9%) or anxiety (14.9%) symptoms. Only 1% of participants reported taking medication for depression. A large proportion of participants reported receiving some support from family and a partner (76.7 and 77.5% respectively).

**Table 1:** Participant characteristics

	Valid n	Mean ± SD	Median (IQR)
***Health behaviours***			
MVPA (hours/week)	1506	10.7 ± 11.8	7 (3–14)
SB (hours/week)	1460	19.0 ± 18.5	12.4 (5.5–28)
TV time (hours/day)	1705	3.9 ± 2.9	3 (2–5)
Screen time (hours/day)	1703	5.9 ± 4.1	5 (2.5–8)
Sleep (hours/night)	1625	9.2 ± 1.9	9 (8–10)
PSQI global score	1624	4.7 ± 2.8	4 (3–6)
		**Frequency (%)**	
Meeting PA guideline	1638	1413 (86.3)	
Meeting sleep duration guideline	1625	779 (47.9)	
Poor quality sleep	1624	543 (33.4)	
***Mental health indicators***			
Depression	1640	310 (18.9)	
Anxiety	1640	245 (14.9)	
Happiness rating^a^	1623		
*1 (unhappy)*		55 (3.4)	
*2*		87 (5.4)	
*3*		447 (27.5)	
*4*		479 (29.5)	
*5 (very happy)*		540 (33.3)	
***Risk factors***			
ACEs score	1268	3.3 ± 2.3	3 (2–5)
Alcohol-use: at risk	1640	397 (24.2)	
SVI: classified as vulnerable	1649	561 (25.4)	
***Protective factors***			
GSE score	1637	30.4 ± 6.3	31 (26–35)
Family support	1637		
*Some support*		1255 (76.7)	
*No support*		382 (23.3)	
Partner support	1465		
*Some support*		1135 (77.5)	
*No support*		330 (22.5)	

*ACEs* Adverse Child Experiences, *GSE* General Self-Efficacy, *MVPA* moderate- to vigorous-intensity physical activity, *PSQI* Pittsburgh Sleep Questionnaire Index, *SB* sedentary behaviour, *SVI* Social Vulnerability Index, *TV* television

^a^0.6% ‘Don’t know’, 0.3% ‘Refuse’

The results of the multiple linear regression analyses are shown in Table 2. Depression (β = 0.161, *p* < 0.001), anxiety (β = 0.126, *p* = 0.001), alcohol-use risk (β = 0.089, *p* = 0.002), and adverse childhood experiences (β = 0.076, *p* = 0.014) were all associated with poorer quality sleep (higher PSQI score). Alcohol-use risk was associated with higher screen time (β = 0.105, *p* < 0.001), lower sitting time, and higher TV time (β = 0.075, *p* < 0.016). Social vulnerability was associated with lower sitting time (β = − 0.187, *p* < 0001) and screen time (β = − 0.014, *p* < 0.001). Higher self-efficacy was associated with higher MVPA (β = 0.07, *p* = 0.036) and better-quality sleep (lower PSQI score, β = − 0.069, *p* = 0.020), whereas lower self-efficacy was associated with higher TV time (β = − 0.079, *p* = 0.012). Having no family support was associated with higher sitting time (β = 0.075, *p* = 0.022).

**Table 2:** Multiple linear regression results for health behaviour outcomes and mental health, social vulnerability, self-efficacy, and social support

	Total MVPA h/wk (***n*** = 968)		Total SB h/wk (***n*** = 928)		Screen time h/day (***n*** = 1070)		TV time h/day (***n*** = 1072)		Global PSQI score (***n*** = 1072)	
	β (95%CI)	*p*	β (95%CI)	*p*	β (95%CI)	*p*	β (95%CI)	*p*	β (95%CI)	*p*
Age	**−0.071 (− 0.70- -0.03)**	**0.033**	**− 0.152 (− 1.80- -0.08)**	**< 0.001**	− 0.027 (− 0.17–0.06)	0.381	0.051 (− 0.01–0.14)	0.102	− 0.042(− 0.13–0.02)	0.154
BMI	0.011 (− 0.09–0.14)	0.741	**− 0.068 (− 0.40- -0.01)**	**0.035**	0.06 (0.00–0.08)	0.051	**0.077 (0.01–0.06)**	**0.013**	− 0.026 (− 0.04–0.01)	0.364
***Mental health indicators***										
Depression (1 = above threshold)	− 0.010 (− 2.70–2.10)	0.809	−0.041 (− 1.80–5.80)	0.309	0.017(− 0.63–1.00)	0.657	0.019 (− 0.41–0.69)	0.617	**0.161 (0.65–1.71)**	**< 0.001**
Anxiety (1 = above threshold)	0.061 (− 0.60–4.80)	0.130	< 0.001 (− 4.30–4.30)	0.999	0.044 (− 0.38–1.42)	0.257	−0.009 (− 0.68–0.53)	0.809	**0.126 (0.44–1.61)**	**0.001**
Happiness rating (1 = unhappy)	− 0.014 (− 5.20–3.40)	0.678	−0.006 (− 7.70–6.30)	−.850	0.004 (− 1.38–1.56)	0.901	0.017 (− 0.73–1.26)	0.597	0.050 (− 0.15–1.76)	0.097
Taking depression meds (1 = yes)	− 0.006 (− 9.60–7.90)	0.848	0.005 (− 10.90–12.80)	0.876	−0.031 (− 4.10–1.30)	0.312	−0.084 (− 4.40–0.74)	0.006	**0.07 (0.41–3.89)**	**0.015**
***Risk factors***										
ACEs score	0.061 (−0.04–0.70)	0.082	0.016 (−0.40–0.70)	0.648	− 0.005 (− 0.13–0.98)	0.881	−0.005 (− 0.08–0.07)	0.868	**0.076 (0.02–0.17)**	**0.014**
Alcohol-use risk (1 = at risk)	0.034 (− 0.80–2.60)	0.303	**0.065 (−5.50- -0.05)**	**0.046**	**0.105 (0.42–1.58)**	**0.001**	**0.075 (0.09–0.87)**	**0.016**	**0.089 (0.21–0.96)**	**0.002**
SVI risk (1 = at risk)	−0.06 (−3.30–0.20)	0.082	**− 0.187 (−10.80- -5.32)**	**< 0.001**	**−0.14 (−1.90- -0.80)**	**< 0.001**	−0.018 (− 0.51–0.27)	0.559	0.011 (− 0.30–0.44)	0.706
***Protective factors***										
GSE score	**0.069 (0.01–0.24)**	**0.036**	0.034 (−0.08–0.30)	0.288	−0.042 (− 0.07–0.01)	0.171	**−0.079 (− 0.06- -0.01)**	**0.012**	**−0.069 (− 0.06- -0.01)**	**0.020**
Support family (1 = no support)	−0.025 (− 1.20–2.60)	0.459	**0.075 (0.50–6.30)**	**0.022**	0.026 (− 0.36–0.87)	0.412	0.005 (− 0.38–0.45)	0.881	0.031 (− 0.19–0.61)	0.305
Support partner (1 = no support)	−0.027 (−2.60–1.10)	0.416	−0.034 (−4.40–1.30)	0.291	−0.024 (− 0.84–0.36)	0.432	−0.018 (− 0.53–0.28)	0.553	0.012 (− 0.30–0.47)	0.674

Significance was accepted at *p* < 0.05 (highlighted in bold). *ACEs* Adverse Child Experiences, *BMI* body mass index, *CI* confidence interval, *GSE* General Self-Efficacy, *MVPA* moderate- to vigorous-intensity physical activity, *PSQI* Pittsburgh Sleep Questionnaire Index, *SB* sedentary behaviour, *TV* television, *SVI* Social Vulnerability Index

The binomial logistic regression results (with adjusted odds ratios ORs) shown in Table 3, support these findings regarding sleep quality. Being classified with depression (OR = 2.036, *p* = 0.003), or anxiety (OR = 2.425, *p* < 0.001), and risk of harmful alcohol-use (OR = 1.419, *p* = 0.028) were all associated with higher odds of being categorised as having poor sleep quality category (X^2^ (12) = 123.158, p < 0.001). However, no other variables were associated with not meeting the sleep duration guideline (X^2^ (12) = 11.14, *p* = 0.517), or the physical activity guideline (X^2^ (12) = 13.39, *p* = 0.342).

**Table 3:** Binomial logistic regression results for health behaviours (outcomes), and mental health, social vulnerability, self-efficacy, social support

	Not meeting PA guideline (***n*** = 968)		Not meeting sleep guideline (***n*** = 1070)		Poor quality sleep classification (***n*** = 1072)	
	Adj OR (95%CI)	*p*	Adj OR (95%CI)	*p*	Adj OR (95%CI)	*p*
Age	1.039 (0.95–1.14)	0.411	1.001 (0.94–1.06)	0.981	0.944 (0.88–1.01)	0.086
BMI	0.988 (0.96–1.02)	0.475	0.986 (0.97–1.01)	0.158	0.999 (9.98–1.02)	0.961
***Mental health indicators***						
Depression (1 = above threshold)	0.842 (0.43–1.64)	0.611	0.864 (0.57–1.30)	0.768	**2.036 (1.28–3.23)**	**0.003**
Anxiety (1 = above threshold)	1.539 (0.77–3.07)	0.221	1.070 (0.68–1.67)	0.481	**2.425 (1.60–3.67)**	**< 0.001**
Happiness rating (1 = unhappy)	0.223 (0.03–1.69)	0.147	1.281 (0.61–2.67)	0.511	2.086 (0.91–4.76)	0.081
Taking depression meds (1 = yes)	3.841 (0.88–16.65)	0.072	1.232 (0.32–4.70)	0.761	**11.08 (1.30–94.12)**	**0.028**
***Mental health risks***						
ACEs score	0.946 (0.86–1.04)	0.244	0.967 (0.91–1.02)	0.253	1.05 (0.98–1.12)	0.139
Alcohol-use risk (1 = at risk)	1.075 (0.68–1.69)	0.754	1.258 (0.94–1.67)	0.118	**1.419 (1.04–1.94)**	**0.028**
SVI risk (1 = at risk)	0.708 (0.43–1.16)	0.167	1.330 (1.00–1.77)	0.052	1.075 (0.78–1.48)	0.657
***Protective factors***						
GSE score	0.983 (0.95–1.01)	0.280	0.992 (0.97–1.01)	0.446	0.980 (0.96–1.0)	0.061
Support family (1 = no support)	1.079 (0.67–1.75)	0.757	0.961 (0.71–1.30)	0.798	1.086 (0.77–1.52)	0.632
Support partner (1 = no support)	1.085 (0.68–1.73)	0.734	1.007 (0.75–1.35)	0.962	1.127 (0.81–1.56)	0.476

Significance was accepted at *p* < 0.05 (highlighted in bold). *ACEs* Adverse Child Experiences, *Adj OR* adjusted odds ratio, *BMI* body mass index, *CI* confidence interval, *GSE* General Self-Efficacy, *MVPA* moderate- to vigorous-intensity physical activity, *PA* physical activity, *PSQI* Pittsburgh Sleep Questionnaire Index, *SVI* Social Vulnerability Index, *TV* television

Neither self-rated happiness nor partner support were found to be associated with any outcome variables in the regression analyses. However, in the bivariate correlations (Supplementary Table 1), a higher happiness rating and the presence of partner support were both associated with lower anxiety and depression risk, a lower ACEs score, a lower PSQI score (better sleep quality), and higher self-efficacy (all *p* < 0.05). Self-rated happiness was also associated with lower TV time (*p* < 0.05). However, these were unadjusted correlations, and the results should be interpreted with caution.

## Discussion

This study contributes to our understanding of the relationships between mental health and physical activity, sedentary behaviour and sleep in young, socially vulnerable South African women from Soweto, a low-income, urban setting. The most significant finding from this study was that numerous indicators of mental health and risk factors related to mental health were associated with poor sleep quality, but not necessarily with meeting the sleep duration guideline, and the findings confirm the phenomenon of long self-reported sleep amongst women in other studies in South Africa [39, 40]. Alcohol-use risk was associated with higher screen and TV time, which could suggest a clustering of risk behaviours. With regards to protective factors, women with higher self-efficacy were more likely to have better sleep quality, but social support did not appear to play a role in meeting the sleep duration guideline or sleep quality. Women with higher self-efficacy were more likely to have higher levels of physical activity, but none of the mental health indicators, nor social support were associated with physical activity levels, or with meeting the physical activity guideline. As would be expected, there was an inverse relationship between self-efficacy and TV time, and similarly between a lack of social support from family and sitting time. Our hypothesis (that poor mental health indicators and risks are barriers to health behaviours of young women in Soweto, and that self-efficacy and social support are protective for health behaviours of young women in Soweto) is therefore only partly confirmed.

These sleep-related findings support previous research, including that conducted in LMICs, that unusually long sleep durations have been reported in other studies with South African women [39, 40] and that poor sleep quality is associated with greater risk for anxiety and depression as well as alcohol-use risk [9, 10, 13, 33–37]. Sleep quality appears to play a more significant role in mental health than meeting sleep duration guidelines for young women in this setting, similar to previous findings from the UK [37]. One might speculate that either past experiences or social and environmental exposures such as poverty or high crime might exacerbate anxiety and depression, which in turn is known to reduce sleep quality. Indeed, young South African women from low-income settings with post-traumatic stress disorder reported poorer sleep quality than those with no prior trauma exposure [72], and the finding in our study that adverse childhood experiences was associated with worse sleep quality among these women supports this line of thinking. The lack of any association between social vulnerability and sleep quality, however, was unexpected. It is possible that poverty-related factors in the SVI (such as assets, education, employment) are less problematic for sleep than factors related to crime or safety. There is evidence to suggest that sleep quality is worse among individuals living in neighbourhoods that are noisy, unclean or have high crime [73], and that the association between neighbourhood disorder and psychological distress is amplified by poor sleep quality [74]. Thus, the poor-quality sleep observed in the present study may exacerbate symptoms of depression and anxiety in these women. In this case, individuals might extend sleep to compensate for both poor sleep quality, and in response to feeling depressed or anxious during the daytime. More research is needed to investigate this further, especially since 44% of the women in this study were long sleepers (> 9 h per night) and one third reported poor sleep quality. Understanding how mental and physical health risks may co-exist in young women who are ‘long sleepers’ and/or not getting quality sleep is of particular interest. It is encouraging that women with higher self-efficacy report better sleep quality, since this suggests that despite circumstances which have the potential to perpetuate poverty, poor mental health and higher risk for NCDs, individual traits such as self-efficacy may be able to limit poor sleep quality and its consequences on physical and mental health. This has implications for the inclusion of self-efficacy in future interventions to promote sleep quality in this population.

Our observations do not align with other research that have examined the converse causality of these relationships, and have indicated the potential protective and curative role of physical activity for mental ill-health [9, 10, 15–17]. Given previous findings in this setting, it is likely that this is context-related [50], and further research is required in this setting to better understand the dose and context of physical activity required to promote mental health. In particular, attention should be paid to leisure-time physical activity, which is typically recommended for mental health benefits, although it implies a degree of autonomy and volition [17, 75], and the element of choice to participate in health behaviours may be limited in settings such as Soweto [76]. Given that there are numerous barriers to this type of physical activity for young women in this setting [50], careful thought should be given to the contextual realities when promoting leisure-time physical activity. The relationships between mental health, and sitting time, screen and TV time also appear less clearly defined. While other studies (again, examining converse causality) have shown that higher sedentary behaviour levels are associated greater mental health risk [8, 11, 23, 24], we only found that at-risk alcohol use was associated with more screen-related sedentary behaviours. Future research is necessary to explore this finding and establish whether these risks are clustered, as well as to determine the direction of these relationships.

Based on the findings of this study, it could be argued that addressing mental health may improve sleep quality, sitting time, screen and TV time amongst young women in this setting. Drawing on family support may be more important than partner support when it comes to these health behaviours. For all these health behaviours, it is likely that there are various broader socioecological factors (e.g., structural violence, infrastructure) that need addressing in order to optimise the relationship between mental (and physical) wellness and health behaviours, many of which have already been identified in previous qualitative work in this setting [46, 50–52]. Additional work is needed to further investigate how these, and other systematic factors could be influencing sleep and other health behaviours amongst young women in this setting. This research should also aim to identify potential entry-points for intervention, taking into consideration that the home environment needs to be considered, and not only individual behaviour change. Despite the presence of a range of challenging systemic factors, it does appear that self-efficacy is an appropriate target for behaviour change strategies in this setting; this is encouraging, given the recognised role of developing self-efficacy in behaviour change interventions [41]. Additionally, young women in LMIC settings can face a range of acute, chronic and intergenerational trauma, which pose a substantial risk to their mental health. This exposure to trauma has emerged in other HeLTI qualitative work with young women participating in the trial (in review) [77]. The influence of trauma on behaviour change [78] is a relatively new area of study, but can provide a meaningful and useful framework for understanding the promotion of health in its broadest sense with young women in these settings.

A limitation of this study is that associations identified are cross-sectional, and causality cannot be inferred. Future longitudinal analyses in the HeLTI trial, which follows participants, and if they become pregnant, their offspring from preconception through to early childhood, will be able to examine the potential bidirectionality of the associations between mental health and sleep quality, e.g., does poor mental health lead to unhealthy behaviours, or do unhealthy behaviours cause poor mental health? Furthermore, this study did not explore the relationships between mental health indicators, risk, and protective factors in detail; this would be a valuable direction for future research. The use of self-reported physical activity, sedentary behaviour and sleep data could also be considered a limitation of this study given the risk for recall bias and the likely overestimation of MVPA, for example, although the GPAQ is among the best available tools for use in large samples such as this study. The use of a number of previously validated and contextually relevant measures of sleep quality and mental health is a strength of this study. Furthermore, while these findings cannot be directly generalised to other low-income settings in South Africa, they provide valuable insights that could be applied in these, and other LMIC settings, as well as in low-income settings in high-income countries.

## Conclusion

In conclusion, given the burden of mental disorders that exist alongside the burden of NCDs in South Africa, these findings, along with previous qualitative work, emphasise the need to further investigate relationships between mental health and health behaviours amongst young women in Soweto. The findings of this study add to previous work that has stressed how interventions, such as the HeLTI trial [51], should aim to optimise both physical and mental health in young women from low-income settings. Given the social vulnerability of women in these settings, interventions should be located within a bio-social life-course approach [79] to take into consideration how these risks, along with psychological, social and economic factors impact on young women’s physical and mental health; and consequently the health and development of the next generation. To do this in a way that is contextually appropriate and effective, it is imperative to understand how mental health challenges and social vulnerabilities are associated with health behaviours. Further work is necessary to better understand these relationships, while taking into consideration existing structural factors and broader contextual realities that are barriers to these behaviours.

## Supplementary Information

**Additional file 1: Supplementary Table 1. **Bivariate correlations.

## Data Availability

The dataset analysed for this study is available from the corresponding author on reasonable request.
