# Supplementary material for: Cross-sectional associations between mental health indicators and social vulnerability, with physical activity, sedentary behaviour and sleep in urban African young women
Source: Int J Behav Nutr Phys Act. 2022 Jul 10;19:82. doi: 10.1186/s12966-022-01325-w (PMC9272865; doi:10.1186/s12966-022-01325-w)
Supplement: Supplementary file 1 — Additional file 1: Supplementary Table 1. Bivariate correlations. [file 12966_2022_1325_MOESM1_ESM.docx]

**Supplementary Table 1:** Bivariate correlations

|  | **1**  **Age** | **2**  **BMI** | **3**  **Dep meds** | **4 Happy rating** | **5**  **Supp part** | **6**  **Supp family** | **7**  **Anx** | **8**  **Dep** | **9**  **Alc risk** | **10**  **SVI risk** | **11**  **ACEs** | **12**  **GSE** | **13**  **MVPA** | **14**  **Sitting time** | **15**  **Screen time** | **16**  **TV time** | **17**  **PSQI** | **18**  **PA GL** | **19**  **SB GL** | **20**  **Slp GL** |
| --- | --- | --- | --- | --- | --- | --- | --- | --- | --- | --- | --- | --- | --- | --- | --- | --- | --- | --- | --- | --- |
| 1 | -- |  |  |  |  |  |  |  |  |  |  |  |  |  |  |  |  |  |  |  |
| 2 | **.203*** | -- |  |  |  |  |  |  |  |  |  |  |  |  |  |  |  |  |  |  |
| 3 | **-.073*** | -0.017 | -- |  |  |  |  |  |  |  |  |  |  |  |  |  |  |  |  |  |
| 4 | 0.012 | 0.03 | -.074* | -- |  |  |  |  |  |  |  |  |  |  |  |  |  |  |  |  |
| 5 | 0.063 | 0.013 | -0.016 | .206* | -- |  |  |  |  |  |  |  |  |  |  |  |  |  |  |  |
| 6 | **.107*** | -0.018 | 0.047 | 0.06 | .107* | -- |  |  |  |  |  |  |  |  |  |  |  |  |  |  |
| 7 | 0.025 | -0.002 | **.121*** | **-.269*** | **-.093*** | 0.034 | -- |  |  |  |  |  |  |  |  |  |  |  |  |  |
| 8 | -0.018 | -0.047 | **.095*** | **-.258*** | **-.148*** | -0.029 | **.594*** | -- |  |  |  |  |  |  |  |  |  |  |  |  |
| 9 | **.118*** | 0.02 | -0.02 | -.070* | -0.01 | 0.014 | 0.06 | **.081*** | -- |  |  |  |  |  |  |  |  |  |  |  |
| 10 | **.096*** | -0.017 | -0.052 | -0.053 | 0.015 | -0.035 | 0.034 | 0.055 | 0.044 | -- |  |  |  |  |  |  |  |  |  |  |
| 11 | 0.025 | -.073* | 0.022 | **-.276*** | **-.212*** | **-.069*** | **.214*** | **.235*** | **.167*** | **.153*** | -- |  |  |  |  |  |  |  |  |  |
| 12 | 0.055 | 0.001 | -0.048 | **.271*** | **.094*** | **.069*** | **-.093*** | **-.111*** | **-.077*** | **-.115*** | **-.075*** | -- |  |  |  |  |  |  |  |  |
| 13 | **-.074*** | 0.013 | 0.005 | 0.044 | -0.038 | 0.014 | 0.037 | 0.016 | 0.043 | -0.058 | 0.067 | 0.059 | -- |  |  |  |  |  |  |  |
| 14 | **-.195*** | -.107* | 0.018 | -0.056 | -.085* | 0.01 | 0.038 | 0.06 | **-.092*** | **-.202*** | 0.002 | 0.039 | .085* | -- |  |  |  |  |  |  |
| 15 | -0.019 | 0.045 | -0.033 | -0.05 | -0.04 | 0.018 | 0.024 | 0.019 | **.094*** | -.150* | 0.001 | **-.080*** | -0.032 | **.117*** | -- |  |  |  |  |  |
| 16 | **.076*** | **.098**** | **-.094*** | **-.078*** | -0.023 | -0.01 | -0.003 | 0.02 | **.109*** | 0.037 | -0.011 | -.105* | 0 | -0.067 | **.304*** | -- |  |  |  |  |
| 17 | -0.058 | -0.044 | **.089*** | **-.210*** | **-.077*** | -0.026 | **.254*** | **.295*** | **.104*** | 0.047 | **.149*** | **-.147*** | 0.031 | 0.049 | **.084*** | **-.068*** | -- |  |  |  |
| 18 | 0.007 | -0.002 | -.090* | 0.016 | -0.022 | -0.021 | -0.005 | 0.011 | 0.007 | 0.031 | 0.014 | -0.011 | **.103*** | 0.054 | 0.022 | 0.032 | 0.005 | -- |  |  |
| 19 | **.103*** | 0.014 | -0.014 | 0.04 | **.073*** | -0.016 | -0.003 | -0.029 | 0.033 | **.147*** | 0.001 | -0.03 | -0.064 | **-.620*** | **-.079*** | 0.019 | -0.035 | **-.095*** | -- |  |
| 20 | -0.002 | -0.002 | -0.009 | 0.011 | -0.025 | -0.007 | 0.029 | 0.028 | -0.013 | -0.061 | 0.047 | 0.02 | -0.033 | **.082*** | **.081*** | 0.056 | **-.113*** | -0.006 | -0.023 | -- |

BMI: body mass index, Dep meds = taking depression medication, Happy rating = happiness rating, Supp part = partner support, Supp family = family support, Anx = anxiety, Dep = depression, Alc risk = alcohol-use risk, SVI risk = social vulnerability risk, ACEs: Adverse Child Experiences, GSE: General Self-Efficacy, MVPA: moderate- to vigorous-intensity physical activity, PSQI: Pittsburgh Sleep Questionnaire Index, PA GL = meeting physical activity guideline, SB GL = meeting sedentary behaviour guideline, Slp GL = meeting sleep guideline
